# Dual-Channel Scale-Adaptive Clutter Suppression with Confidence-Derived Gating for Microwave Inspection of Curved Radar-Absorbing Materials

**DOI:** 10.3390/s26185854

**Published:** 2026-09-16

**Authors:** Yang Fang, Jiaqi Wang, Wei Cui, Xiaolong Wei, Bin Liu, Yong Li, Zhenmao Chen

**Affiliations:** 1 State Key Laboratory for Strength and Vibration of Mechanical Structures, Shaanxi Engineering Research Center of NDT and Structural Integrity Evaluation, School of Aerospace Engineering, Xi’an Jiaotong University, Xi’an 710049, China; 1163011740@stu.xjtu.edu.cn (J.W.); yong.li@mail.xjtu.edu.cn (Y.L.); chenzm@mail.xjtu.edu.cn (Z.C.); 2 National Key Laboratory of Electromagnetic Information Control and Effects, Shenyang 110037, China; 3 Science and Technology on Plasma Dynamics Laboratory, Air Force Engineering University, Xi’an 710000, China; wei18892022001@163.com; 4 Shaanxi Special Equipment Inspection and Research Institute, Xi’an 710049, China; 19991202881@163.com

**Keywords:** radar-absorbing materials, surface-damage detection, microwave imaging, clutter suppression, semantic segmentation, confidence-derived gating

## Abstract

Microwave imaging is promising for non-contact inspection of radar-absorbing materials (RAMs); however, strong structured non-damage responses in reconstructed microwave images can resemble surface defects and trigger false detections, while hard masking may remove weak damage responses near ambiguous boundaries. To address these issues, this paper proposes dual-channel scale-adaptive clutter suppression with confidence-derived gating as an application-specific front end for surface-damage detection in curved RAM specimens. A normalized microwave-response map and a fixed morphology-enhanced map, both deterministically derived from the same measurement, are jointly encoded. The input-conditioned scale-adaptive pyramid-pooling module (SA-PPM) reweights multi-scale context branches, while confidence-derived adaptive-threshold gating (C-ATG) generates a bounded pixel-wise threshold from the retention confidence and its ambiguity proxy. Continuous-weight filtering (CW-FI) then produces continuous retention weights, enabling gradual attenuation instead of forced binary removal. Experiments were conducted on a paired visible-light/microwave RAM dataset. On the held-out test subset, the method achieved pixel-level discrimination between clutter and retained responses and improved detection of strip-like cracks and circular spalling under matched detector-side settings.

## 1. Introduction

Radar-absorbing materials (RAMs) reduce reflected and scattered electromagnetic energy and are widely used in radar-stealth and electromagnetic-protection applications [1]. Recent reviews show that absorption performance is governed jointly by material composition, microstructure, impedance matching, and structural design [1,2]. In service, impact damage can change the return loss and electromagnetic performance of radar-absorbing composite structures, motivating reliable condition assessment and maintenance [3].

Free-space measurements provide non-contact characterization of reflectivity and dielectric properties, but their accuracy depends on calibration, incidence angle, standoff distance, sample geometry, and the measurement environment [4,5]. Microwave imaging offers a spatially resolved alternative for inspection, while near-field multiple-input multiple-output (MIMO) and synthetic-aperture radar (SAR) methods accommodate irregular array layouts and non-ideal scanning trajectories [6,7].

Sparse MIMO-array synthesis can reduce the number of physical antenna elements while controlling near-field imaging performance [8]. Near-field MIMO-SAR studies further address scanning-array compensation [9], efficient range-migration processing [10], regularized reconstruction [11], and physics-based learned reconstruction from compressive measurements [12]. Together with irregular-array and irregular-trajectory methods [6,7], these developments provide practical alternatives for microwave image formation under constrained sampling.

The microwave response of RAM is jointly influenced by material composition, microstructure, interfaces, and measurement geometry [1,2]. Variations in material structure can alter local electromagnetic parameters; curvature can modify surface normals, incidence conditions, and propagation paths; and scanning geometry affects the superposition of different scattering contributions. Consequently, a ring-like, patch-like, or strip-like appearance does not uniquely determine the physical origin of the response; therefore, this paper adopts a detection-task-oriented definition of clutter.

Reference [13] established a clutter suppression workflow for RAM microwave images based on PSPNet semantic segmentation, utilizing a MobileNetV2 backbone and standard pyramid pooling, and demonstrated the advantages for downstream detection through ablation studies comparing results with and without clutter suppression. Building upon this foundation, the present paper further investigates how multi-scale context adapts to the input and how segmentation confidence is converted into retention weights for detection. SA-PPM adjusts scale-branch weights based on input features; C-ATG generates bounded pixel-wise thresholds by combining an image-level base threshold with a confidence-derived ambiguity proxy; and CW-FI converts retention confidence into continuous weights that are applied to the raw microwave response map. The primary contributions of this paper in this regard lie in the coordinated design of these processing stages and the controlled evaluation of their impact on downstream detection.

Despite improvements in acquisition and reconstruction, structured clutter remains a major limitation for automatic damage detection. In microwave RAM images, clutter caused by scattering coupling and non-uniform absorber distribution can exhibit strong responses resembling damage-related features [13]. Multi-scale context modeling has also proved useful for semantic segmentation of structured SAR scenes [14]. Recent object-detection reviews describe the efficiency of one-stage detectors [15], with the real-time one-stage design in Ref. [16] providing a representative architecture; broader and defect-oriented reviews further document the speed–accuracy trade-off of these detector families [17,18]. However, such detectors do not explicitly remove structured clutter from the input. This study therefore focuses on front-end semantic segmentation and clutter suppression before damage detection.

At the interface between image formation and detection, different treatments of array irregularity, range migration, regularization, and learned priors can alter reconstruction artifacts and the apparent morphology of target responses [6,7,8,9,10,11,12]. The detector therefore receives acquisition- and reconstruction-dependent morphologies. This variability further motivates an input-side suppression stage whose effect is assessed without changing detector-side settings within each comparison.

Pixel-level segmentation provides explicit regional assignments and defect outlines that are suitable for front-end clutter suppression; recent industrial-inspection studies compare detection with semantic segmentation [19] and demonstrate efficient segmentation-based surface-defect analysis [20]. Reviews of efficient convolutional neural network (CNN) structures, architecture optimization, acceleration, and machine-learning optimization provide practical foundations for the lightweight encoder and training protocol [21,22,23,24]. Building on these foundations, the proposed method couples scale-adaptive context modeling with confidence-derived gating and evaluates its front-end contribution under controlled detector configurations.

Taken together, the cited studies establish the value of pixel-level regional modeling for industrial defect analysis [19,20], but near-field microwave-imaging research still emphasizes acquisition and reconstruction, while generic segmentation and detection methods do not explicitly distinguish damage-related responses from strong non-damage structures. In addition, hard masks can remove weak responses near uncertain boundaries. These limitations motivate the dual-channel, scale-adaptive, and continuous-gating design developed below.

Accordingly, this work develops an application-specific front-end clutter-suppression framework that integrates two deterministic views derived from a single microwave-response map, input-conditioned multi-scale context aggregation, and confidence-derived adaptive retention weighting. Its methodological novelty lies in the coordinated formulation of these components and their controlled downstream evaluation for surface-damage detection in curved RAM specimens.

The specific contributions are summarized as follows:

(1) A front-end clutter-suppression framework is established for microwave surface-damage detection in curved RAM specimens, linking pixel-level clutter modeling to a controlled downstream evaluation.
(2) A dual-channel representation combines a normalized microwave-response map with a morphology-enhanced map derived from the same measurement to support discrimination between structured non-damage responses and damage-related responses.
(3) A scale-adaptive pyramid-pooling module (SA-PPM) reweights multi-scale context branches according to the encoded input feature.
(4) Confidence-derived adaptive-threshold gating (C-ATG) and continuous-weight filtering (CW-FI) convert retention confidence into bounded pixel-wise thresholds and retention weights. Their contributions are examined through component ablation and matched downstream detector retraining.

The remainder of this paper is organized as follows: Section 2 introduces the data, annotations, data splits, and evaluation metrics; Section 3 presents the dual-channel representation, scale-adaptive context aggregation, and confidence-derived gating; Section 4 details parameter selection, detection comparisons, and ablation results; Section 5 discusses the results and scope of applicability; and Section 6 concludes the paper.

## 2. Dataset Construction and Problem Definition

This section describes the paired visible-light/microwave data, annotation conventions, training-data augmentation, clutter-suppression task, and evaluation definitions. Visible-light images are used only as offline references for the two annotated surface-damage categories; the model input and downstream evaluation use microwave images.

### 2.1. Microwave Measurement System and Paired Data Acquisition

Microwave response maps of curved RAM specimens were acquired using the near-field multiprobe scattering-imaging system reported for RAMDD [13]. The array operated in spot-frequency scanning mode over 4–18 GHz. A two-dimensional cross-array configuration was used, and the transmit–receive data were reconstructed with a bistatic range-migration algorithm that separates the transmitter and receiver spectra instead of replacing each pair with a single equivalent phase center [13]. The reconstructed response maps were standardized to 416 × 416 pixels.

**Figure 1:**
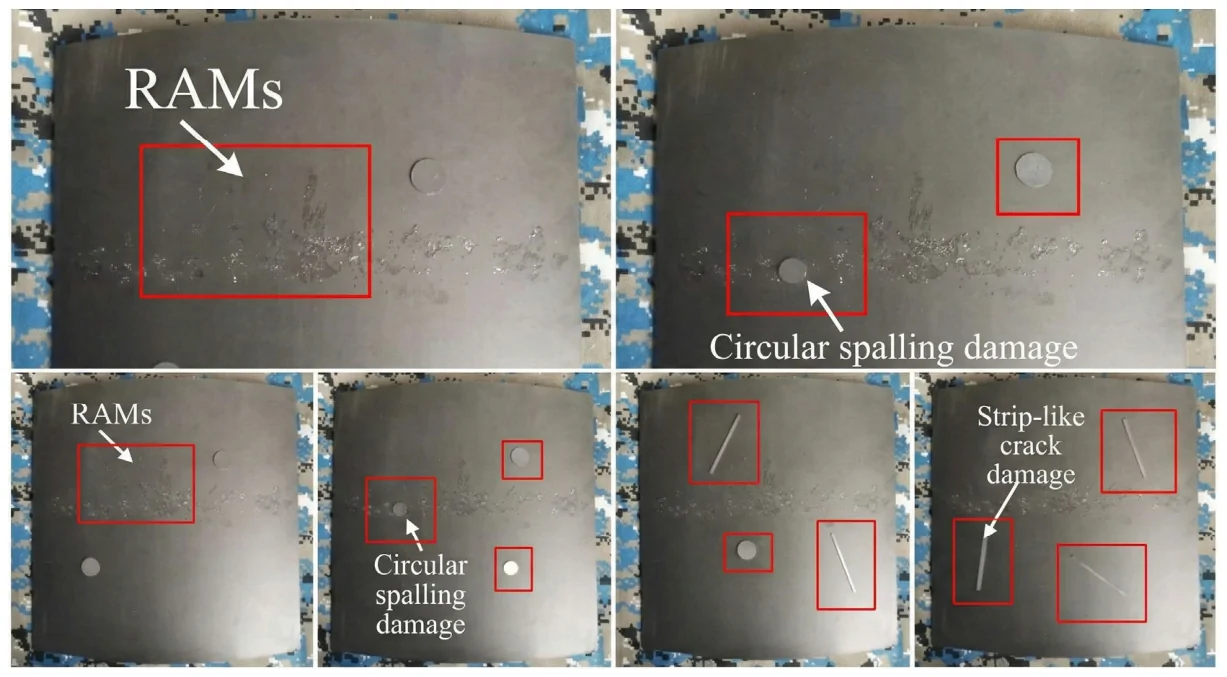
Curved RAM specimens and representative surface-damage morphologies.

**Figure 2:**
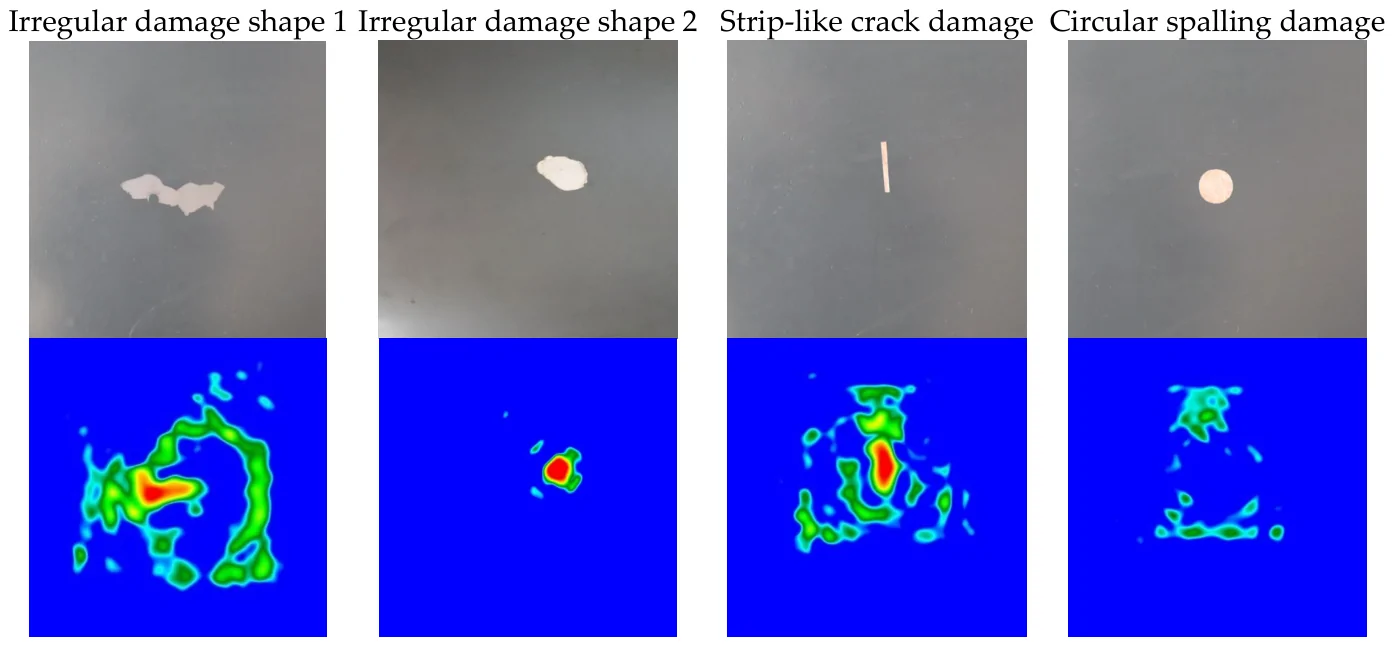
Examples of paired visible-light references (**top row**) and microwave-response maps (**bottom row**).

Corresponding visible-light photographs were acquired from a fixed position and angle for the same specimen regions. They were used to locate visible surface damage and pair each microwave-response map with its annotation reference; Figure 1 and Figure 2 show representative specimens and paired samples.

Clutter segmentation, front-end suppression, and detection-gain evaluation use the microwave image I_mw as the sole model input. The visible-light image I_vis is used only for annotation reference and qualitative interpretation; no visible-light feature is used during training or inference. The pairing is therefore an annotation and traceability mechanism, not a dual-modal fusion architecture.

Visible-light images provide clearer geometric references for locating visible surface damage and help annotators distinguish damage-corresponding microwave responses from strong non-damage responses. They are used only for annotation and traceability and are not provided to the model.

In this paper, clutter is distinguished from retained responses based on the correspondence between microwave responses and two categories of visible surface damage annotations rather than judging damage solely by the appearance of the responses. Visible-light references cannot rule out subsurface anomalies that are not identified by surface annotations; therefore, the conclusions are limited to the specific categories of visible surface damage evaluated.

All images were standardized to 416 × 416 pixels. This study used 1577 visible-light/microwave image pairs containing 3065 instances of circular spalling and 3172 instances of strip-like cracks, totaling 6237 annotated damage instances. Image pairs, physical specimens, and measurement sessions are distinct counting units. Reference [13] reported 1448 image pairs; the difference of 129 pairs does not establish the number of additional independent measurements.

### 2.2. Damage and Clutter Annotation Definitions

Two annotation types are used: damage-instance bounding boxes for downstream detection and pixel-level clutter/retained-response labels for front-end segmentation. Visible-light images provide references for surface-damage localization, with strip-like cracks annotated using their minimum bounding rectangles. Visible-light and microwave images are paired by physical specimen and scan region; this pairing does not imply pixel-wise alignment between visible-light damage contours and microwave-response boundaries. The detection categories are strip-like cracks and circular spalling, and visible-light images are not used as model inputs. The labels “Irregular damage shape 1” and “Irregular damage shape 2” in Figure 2 describe example morphologies; they neither define an additional detection category nor determine the clutter labels.

For clutter annotation, clutter localization is formulated as binary pixel classification, with class 0 denoting clutter and class 1 denoting a retained response (the operational non-clutter class). The corresponding ground-truth retention mask is $M_{ret,gt}(i)=I[y_{gt}(i)=1]$. Regions without corresponding annotated visible surface damage but with localized strong microwave responses are labeled as clutter under the operational definition above. Ambiguous boundaries were labeled conservatively to reduce the risk that damage-consistent responses would be assigned to the clutter class and subsequently suppressed; label uncertainty was not modeled explicitly.

### 2.3. Front-End Segmentation Training Data Augmentation

The dataset used in the partitioning process described herein comprises 1577 visible-light/microwave image pairs. Grouping was performed based on the physical specimens; the training, validation, and test sets contain 1261, 158, and 158 image pairs, respectively, accounting for 79.96%, 10.02%, and 10.02% of the total. Data from the same specimen—including overlapping or adjacent scan areas—were assigned to the same subset. The geometric and Mosaic augmentations described in this paper were applied exclusively to the training set after partitioning; no such augmentations were performed during the validation and test phases. Spatial transformations were applied synchronously to the microwave images, their annotations, and their paired references, with the same data split utilized for both the upstream segmentation and downstream detection tasks. Visible-light images were used solely for offline annotation and sample correspondence, while model training and evaluation relied exclusively on microwave data.

**Figure 3:**
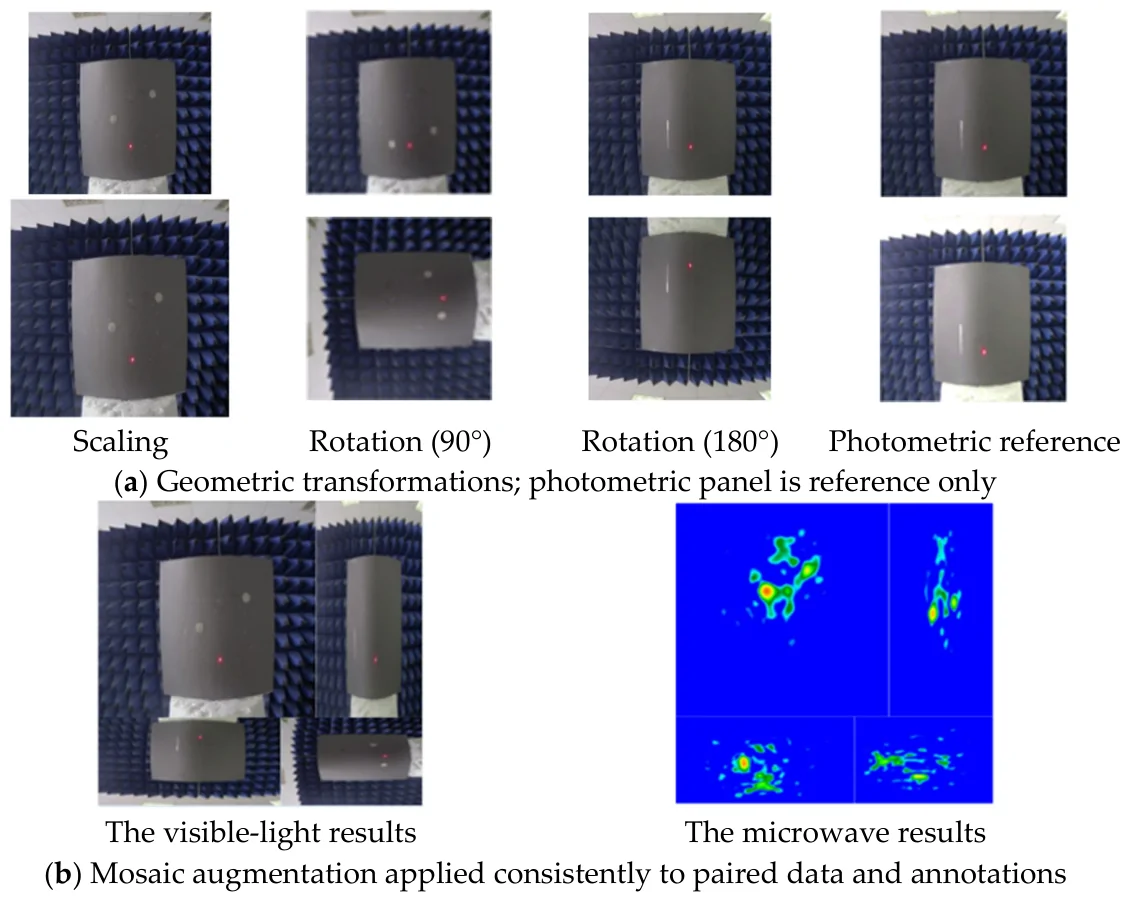
Examples of training data augmentation. (**a**) Geometric augmentation includes scaling by a factor of 0.8–1.2, rotation by 90°, 180°, or 270°, and horizontal or vertical flipping with a probability of 0.5; photometric variations are shown for reference only. (**b**) Mosaic combines four training samples. Microwave images, pixel masks, damage bounding boxes, and visible-light references use identical geometric transformations and stitching layouts; masks are resampled using nearest-neighbor interpolation. Visible-light images are used only for offline annotation and traceability. These augmentations are applied only to the training set.

For geometric augmentation, the operations include random scaling with s∼U(0.8,1.2), discrete clockwise rotations of 90°, 180°, or 270°, and horizontal or vertical flipping with a probability of 0.5. The same spatial transform is applied to the microwave image and its annotations. Class masks require nearest-neighbor interpolation, whereas continuous-valued images use image interpolation. Representative results are shown in Figure 3a.

Mosaic combines four training samples and their annotations to enhance the diversity of scenes and damage instances; no additional independent random cropping is applied, thereby minimizing the truncation of small damage areas and the loss of paired information. As the extent of microwave responses may differ from that of visible-light damage, sharing transformation parameters does not imply pixel-level boundary alignment. Microwave images, pixel masks, damage bounding boxes, and visible-light reference images utilize identical geometric parameters and stitching layouts.

### 2.4. Clutter Segmentation and Front-End Suppression

The front-end task comprises pixel-level clutter segmentation followed by selective suppression of the original microwave response. The segmentation output is converted to either a binary retention mask or a continuous retention-weight map, and the resulting microwave image is supplied to the downstream detector.

Given a microwave image $I_{mw}\in R^{H\times W}$, the segmentation network $f_{\theta }(\cdot )$ outputs the logit vector $z(i)={\left[z_{0}(i),z_{1}(i)\right]}^{T}$ for each pixel i. Class 0 denotes clutter, and class 1 denotes a retained response. The posterior probability is:

(1) $$P_{k}(i)=\frac{exp(z_{k}(i))}{\sum_{j=0}^{1}exp(z_{j}(i))},k\in \{0,1\},$$

where $P_{0}(i)$ is the clutter probability, and $P_{1}(i)=1-P_{0}(i)$ is the predicted retention probability. The latter is used below as the retention-confidence score that drives the gate.

For a fixed threshold τ, the binary retention mask is defined as:

(2) $$M_{ret}(i)=I[P_{1}(i)\geq \tau ],$$

where I denotes the indicator function. The framework supports a binary hard gate generated by thresholding and a continuous-weight gate generated from the retention confidence.

The suppression operator applies pixel-wise retention or attenuation to the original microwave image $I_{mw}$. For the binary hard-gating case, the output is:

(3) $$I_{mw}^{sup}=I_{mw}⊙M_{ret},$$

where ⊙ denotes element-wise multiplication. A value of $M_{ret}(i)=1$ retains the response at pixel i, whereas $M_{ret}(i)=0$ suppresses a response predicted as clutter. This notation avoids conflating clutter labels with retention masks.

Front-end suppression is intended to reduce structured non-damage responses that can trigger false candidates while retaining responses consistent with the annotated damage categories. Its effect is evaluated without changing the detector architecture within each controlled comparison pair.

### 2.5. Segmentation and Class-Wise Detection-Gain Metrics

For segmentation evaluation, mean intersection over union (mIoU) measures agreement between predicted and ground-truth pixel classes. For K classes:

(4) $$mIoU=\frac{1}{K}\sum_{k=0}^{K-1}{IoU}_{k};{IoU}_{k}=\frac{\left|{\hat{\Omega }}_{k}\cap \Omega_{k}^{gt}\right|}{\left|{\hat{\Omega }}_{k}\cup \Omega_{k}^{gt}\right|},$$

where the predicted label is $\hat{y}(i)={argmax}_{k\in K}P_{k}(i)$; ${\hat{\Omega }}_{k}=\{i\in \Omega :\hat{y}(i)=k\}$ is the predicted pixel set; and $\Omega_{k}^{gt}=\{i\in \Omega :y_{gt}(i)=k\}$ is the ground-truth pixel set. |·| denotes set cardinality.

For downstream detection evaluation, detector architecture, specimen-level data split, initialization, training schedule, hyperparameters, and inference post-processing were matched within each comparison pair. Separate detector instances were trained using either the raw microwave-response maps or the full CW-FI-suppressed microwave-response maps. Detection change is reported using class-wise average precision at IoU=0.50 (Standard Average Precision, AP@0.50), which summarizes ranking and localization at the stated IoU threshold.

(5) $$\Delta {AP}_{d}@0.50={AP}_{d,sup}@0.50-{AP}_{d,raw}@0.50,$$

where d denotes a downstream damage category, and the subscripts “sup” and “raw” identify detectors trained on suppressed and raw inputs, respectively. This matched retraining protocol estimates the effect of input-side suppression while avoiding a distribution mismatch between detector training and inference within each comparison pair.

Two failure modes are relevant to front-end suppression: clutter that is incorrectly retained may leave pseudo-responses that trigger false candidates, whereas suppression of retained-response pixels may weaken damage evidence and increase missed detections or localization instability. Because mIoU does not distinguish these downstream consequences, it is reported jointly with class-wise AP@0.50.

## 3. Methodology

The evaluation pipeline comprises pixel-level segmentation, retention-weight generation, suppression of the original microwave-response map, and matched retraining of the downstream detector.

**Figure 4:**
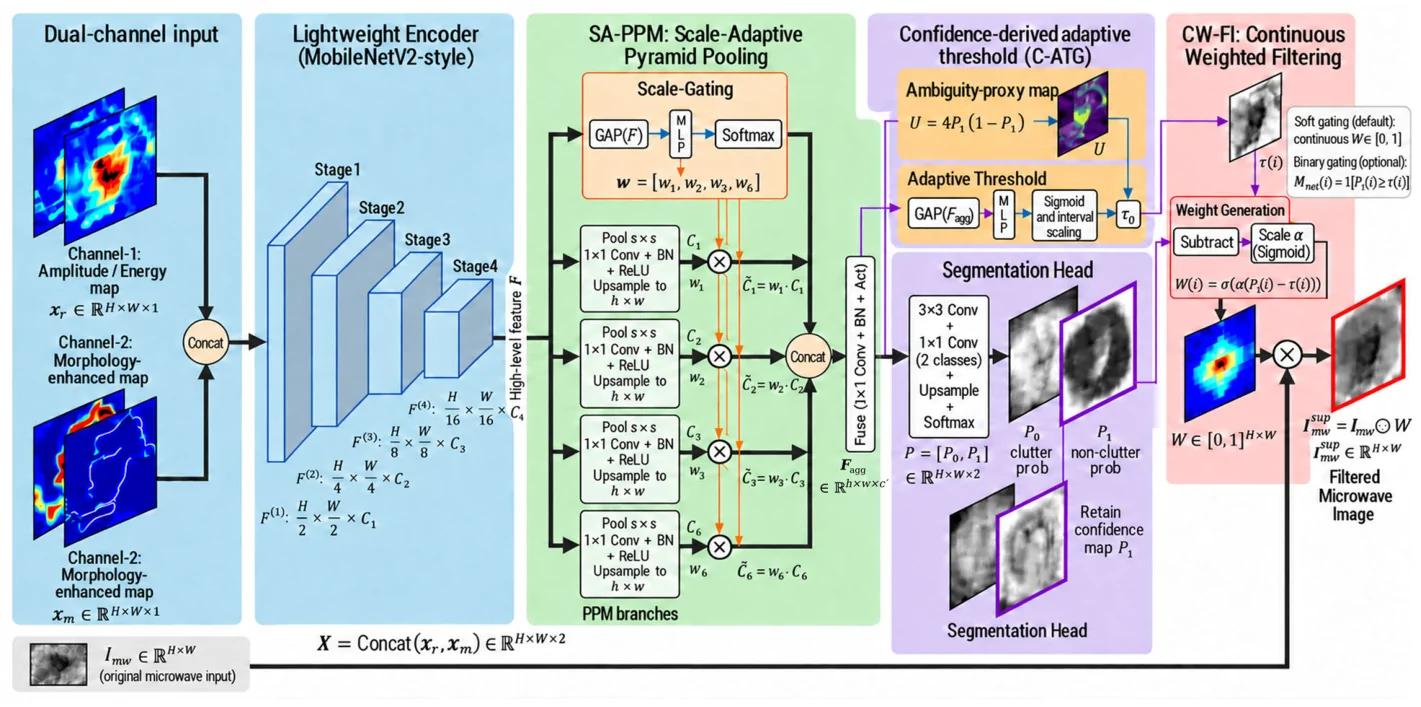
Front-end clutter suppression framework. $x_{r}$ and $x_{m}$, generated from the same microwave measurement, are concatenated to form a dual-channel input X. $F^{\left(l\right)}$ represents the encoded features at the ℓ-th level, while $C_{s}$, $w_{s}$, and $F_{agg}$ denote scale context, scale weights, and fused features, respectively. The segmentation head outputs the clutter probability $P_{0}$ and retention confidence p, which are used to generate W or $M_{ret}$; these are then applied to weight the original single-channel response $I_{mw}$, yielding the detector input $I_{mw}^{sup}$.

The method builds on PSPNet-style context aggregation [25] and combines a dual-channel microwave representation, a MobileNetV2-style encoder, SA-PPM, C-ATG, and CW-FI. The segmentation branch predicts retention confidence, which is converted to binary or continuous weights applied to the original microwave image before detection (Figure 4).

The dual-channel input is defined as:

(6) $$x_{r}=N(I_{mw}),x_{m}=N(T_{m}(I_{mw})),X=Concat(x_{r},x_{m})\in R^{H\times W\times 2},$$

where N(·) denotes the fixed input-normalization operator, $T_{m}(\cdot )$ denotes the fixed morphology-enhancement transform, and Concat denotes channel-wise concatenation. The two channels consist of the raw microwave-response map and the morphological gradient map; these are stacked following frame-by-frame and channel-wise min–max normalization to form a 416 × 416 × 2 input for segmentation. The morphology-enhanced channel does not introduce an independent modality; its gradient is calculated as the difference between a single dilation and a single erosion using a 3 × 3 flat square structuring element, with boundary handling via mirror padding. Visible-light images are not used as network inputs. Identical processing rules are applied during training, validation, and testing.

The binary class-index set is:

(7) K={0,1},

where class 0 denotes clutter to suppress, and class 1 denotes a retained response.

For each pixel, the network produces the logit vector:

(8) $$z(i)={\left[z_{0}(i),z_{1}(i)\right]}^{T},i\in \Omega ,$$

where Ω is the image-pixel set.

Using the logits in Equation (8), the posterior probabilities are computed by Equation (1) for k∈K. Here, $P_{1}(i)$ is the predicted retention probability, $p(i)≜P_{1}(i)$ is the retention-confidence score used by the gate, and $P_{0}(i)=1-P_{1}(i)$ is the clutter probability.

The gate G is constructed from the probability map and applied to the original microwave image:

(9) $$I_{mw}^{sup}=I_{mw}⊙G,$$

where $G\in \{M_{ret},W\}$ denotes either the binary retention mask or the continuous retention-weight map. The detector receives the suppressed single microwave-response map defined by Equation (9), not the dual-channel tensor X used internally by the segmentation network.

### 3.1. Dual-Channel MobileNetV2-Style Encoder

A MobileNetV2-style encoder [26] hierarchically encodes the dual-channel input X and produces multi-scale features for context aggregation:

(10) $$F^{\left(l\right)}\in R^{h_{l}\times w_{l}\times c_{l}},l\in \{1,2,3,4\}.$$

**Figure 5:**
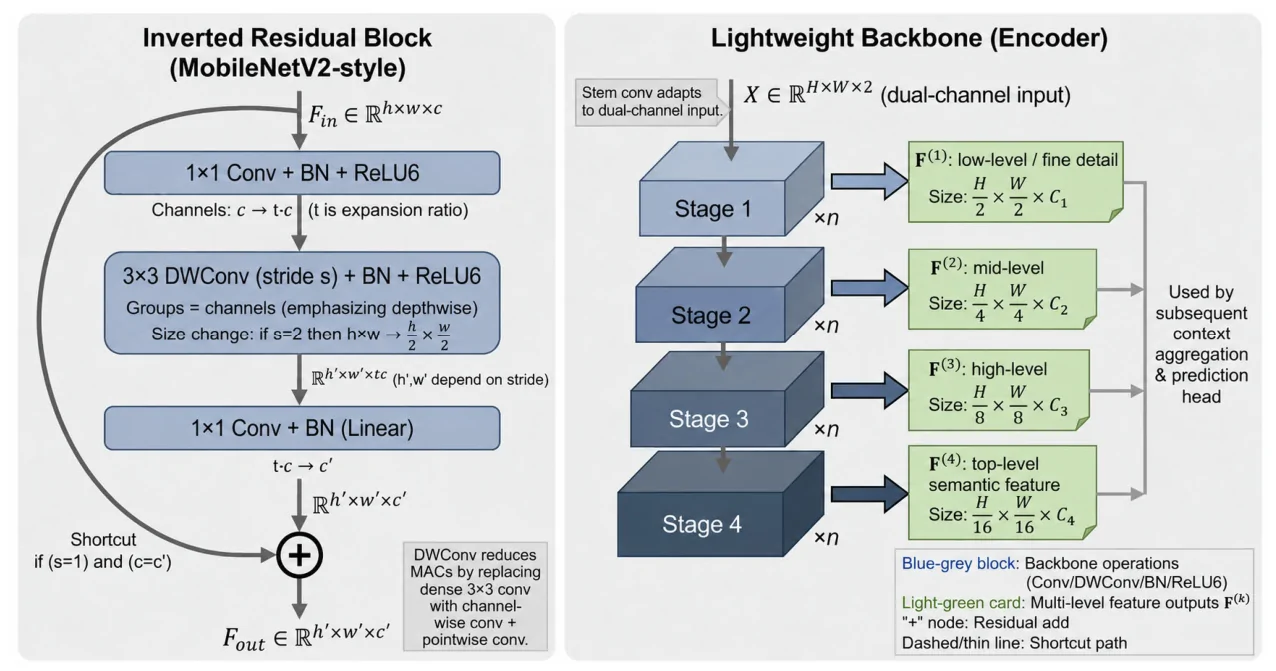
Inverted residual unit and multi-level feature outputs of the MobileNetV2-style encoder.

Shallow features emphasize edges, thin strips, and localized strong responses, whereas deeper features encode regional context. Only the stem convolution is adapted to accept two channels; subsequent inverted residual units follow the MobileNetV2-style structure shown in Figure 5.

To illustrate the computational advantage of lightweight units, let $C_{in}$ and $C_{out}$ denote the input and output channel counts, $D_{k}\times D_{k}$ the convolution-kernel size, and $D_{f}\times D_{f}$ the feature-map size. The multiply–accumulate (MAC) count of a standard convolution is approximated as:

(11) $${Cost}_{std}={D_{k}}^{2}\cdot C_{in}\cdot C_{out}\cdot {D_{f}}^{2}.$$

For a depthwise-separable convolution composed of a depthwise spatial convolution and a 1 × 1 pointwise convolution, the two operation counts are:

(12) $${Cost}_{dw}={D_{k}}^{2}\cdot C_{in}\cdot {D_{f}}^{2},{Cost}_{pw}=C_{in}\cdot C_{out}\cdot {D_{f}}^{2}$$

Thus, the total depthwise-separable cost and its ratio to the standard-convolution cost are:

(13) $${Cost}_{sep}={Cost}_{dw}+{Cost}_{pw},\frac{{Cost}_{sep}}{{Cost}_{std}}=\frac{1}{C_{out}}+\frac{1}{{D_{k}}^{2}}.$$

Figure 5 identifies the encoder units and output stages used by the subsequent context-aggregation and prediction modules. Equation (13) compares one idealized depthwise-separable convolution with a standard convolution of matched spatial dimensions; it excludes the expansion and projection layers of a complete inverted residual unit. It therefore motivates, but does not quantify, end-to-end latency, memory, parameter count, or MACs for the proposed system.

### 3.2. Feature Normalization by Batch Normalization

Batch normalization (BN) follows each convolution unless otherwise specified [27]. It reduces differences in feature scale during optimization and is followed by the stated activation function.

For one feature channel, let ${\left\{a_{i}\right\}}_{i=1}^{m}$ denote the activations collected across the mini-batch and spatial dimensions, with $m=N_{b}h_{f}w_{f}$, where $N_{b}$ is the batch size, and $h_{f}\times w_{f}$ is the feature-map size. BN computes the batch mean and variance as:

(14) $$\mu_{B}=\frac{1}{m}\sum_{i=1}^{m}a_{i},{\sigma_{B}}^{2}=\frac{1}{m}\sum_{i=1}^{m}{\left(a_{i}-\mu_{B}\right)}^{2}.$$

Equation (14) summarizes the channel-wise batch statistics.

Subsequently, each sample is normalized:

(15) $${\hat{a}}_{i}=\frac{a_{i}-\mu_{B}}{\sqrt{{\sigma_{B}}^{2}+\varepsilon_{BN}}},$$

where $\varepsilon_{BN}$ is the positive numerical-stability constant used by batch normalization.

Equation (15) places activations on a comparable scale. A learnable affine transformation then restores representational flexibility:

(16) $$y_{i}=\gamma {\hat{a}}_{i}+\beta ,$$

where γ and β are learnable per-channel scale and shift parameters, respectively, enabling the network to recover appropriate feature amplitudes and offsets while maintaining stable optimization.

During inference, running mean and variance accumulated during training replace the mini-batch statistics.

### 3.3. Scale-Adaptive Pyramid Pooling Module

Structured microwave clutter appears at different spatial scales, including ring-like enclosures, patches, isolated points, and bands. The baseline pyramid-pooling path concatenates its branch outputs without input-dependent scale weights. SA-PPM instead predicts scale weights from the encoded feature. Because this feature is produced jointly from the response and morphology-enhanced channels, the weights are indirectly conditioned on morphology; no explicit area, perimeter, or orientation descriptors are used.

**Figure 6:**
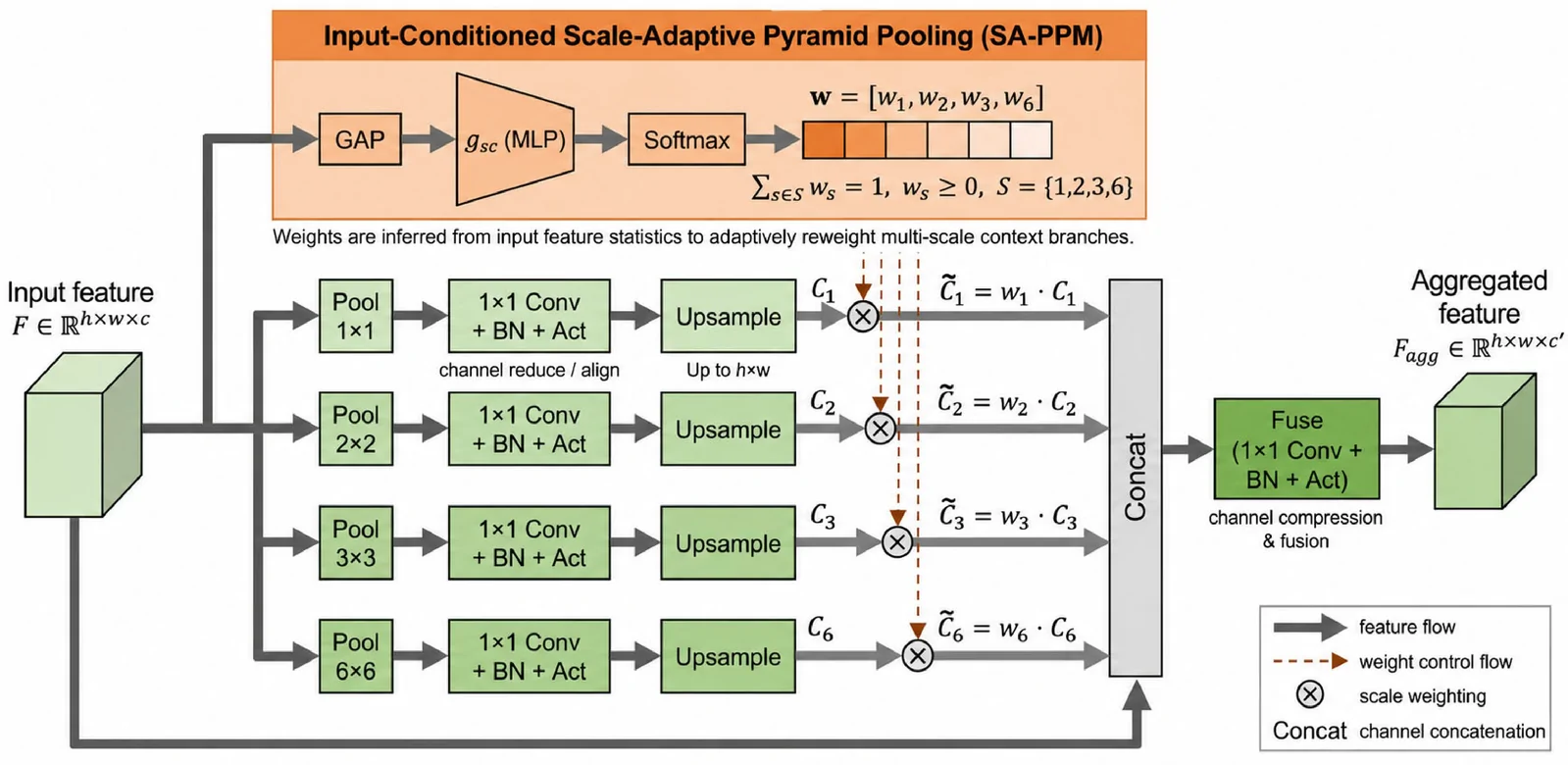
Architecture of the input-conditioned scale-adaptive pyramid-pooling module (SA-PPM).

Let the high-level backbone feature be $F=F^{\left(4\right)}\in R^{h\times w\times c}$. The four pooling grids shown in Figure 6 define S={1,2,3,6}. The contextual output of scale branch s is denoted $C_{s}$:

(17) $$C_{s}=Up(\varphi_{s}({Pool}_{s}(F))),s\in S,$$

where ${Pool}_{s}$ denotes adaptive average pooling to an s×s grid, $\varphi_{s}$ denotes the branch convolution, and Up restores the feature to h×w.

A scale-weight vector $w\in R^{4}$ is predicted from the global descriptor q:

(18) $$q=GAP\left(F\right),w=Softmax\left(g_{sc}\left(q\right)\right),w_{s}\geq 0,\sum_{s\in S}w_{s}=1,$$

where GAP denotes global average pooling, and $g_{sc}$ is the scale-weight prediction network; this notation distinguishes it from the morphology transform $T_{m}$. The softmax operation produces non-negative branch weights that sum to one:

(19) $${\tilde{C}}_{s}=w_{s}\cdot C_{s},s\in S$$

Finally, the backbone feature and the weighted branch contexts ${\tilde{C}}_{s}$ are fused to yield the aggregated feature:

(20) $$F_{agg}=Fuse\left(Concat\left(F,{\tilde{C}}_{1},{\tilde{C}}_{2},{\tilde{C}}_{3},{\tilde{C}}_{6}\right)\right).$$

Fuse(·) uses channel-compression convolution followed by normalization and activation and produces $F_{agg}\in R^{h\times w\times c^{\prime }}$. Equations (17)–(20) define input-conditioned aggregation over the four displayed pooling grids without introducing explicit handcrafted morphology descriptors.

### 3.4. Segmentation Head and Confidence-Derived Adaptive Filtering

C-ATG and CW-FI convert the retention-confidence score into a bounded ambiguity proxy, a pixel-wise threshold, and either continuous or binary retention weights. The formulation balances residual-clutter attenuation against abrupt removal near ambiguous boundaries.

Using the aggregated feature $F_{agg}$, the segmentation head outputs a two-dimensional logit vector at each pixel:

(21) $$L\left(i\right)={\left[H_{seg}\left(F_{agg}\right)\right]}_{i}\in R^{2},$$

where the head uses local convolutions followed by a 1 × 1 projection to two classes. The probability vector follows Equation (1), and the retention-confidence score is $p(i)≜P_{1}(i)$.

To quantify decision ambiguity, a deterministic confidence-derived ambiguity proxy is computed from the single softmax output. It is not an independent Bayesian or model-uncertainty estimate:

(22) $$U\left(i\right)=4p\left(i\right)\left(1-p\left(i\right)\right),$$

where the proxy reaches U(i)=1 at p(i)=0.5 and approaches zero as p(i)→0 or p(i)→1. It is a normalized Bernoulli-variance-like ambiguity score derived from one softmax output. A confidently incorrect prediction also receives a low value, so the proxy does not measure epistemic uncertainty or guarantee calibration.

Using this proxy, a sample-dependent base threshold is predicted from the aggregated feature:

(23) $$\tau_{0}=\tau_{min}+\left(\tau_{max}-\tau_{min}\right)\sigma \left(h_{\tau }\right),$$

where $h_{\tau }=H_{\tau }(GAP(F_{agg}))$ is the scalar output of the threshold branch, and σ denotes the sigmoid function.

**Figure 7:**
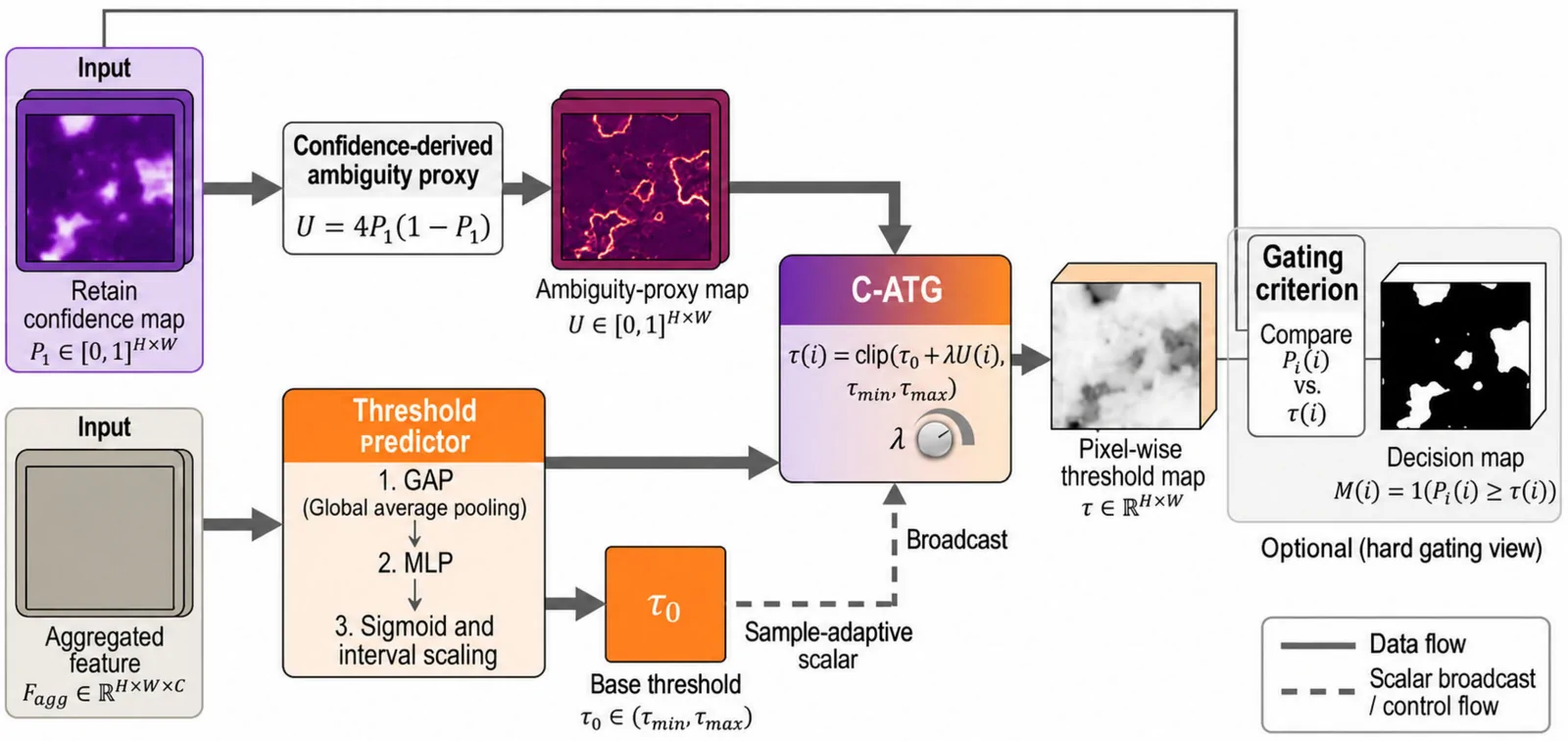
Confidence-derived adaptive-threshold gating (C-ATG). The retention confidence p determines the deterministic ambiguity proxy U, while the aggregated feature determines the image-level base threshold $\tau_{0}$. The bounded pixel-wise threshold τ(i) is compared with p(i) to generate the optional binary retention mask $M_{ret}$. U is not an independent Bayesian or epistemic uncertainty estimate.

In the C-ATG process illustrated in Figure 7, the pixel-wise threshold is then bounded to the configured operating interval:

(24) $$\tau (i)=clip(\tau_{0}+\lambda U(i),\tau_{min},\tau_{max}),\lambda \geq 0,$$

where $0\leq \tau_{min}<\tau_{max}\leq 1$ define the lower and upper bounds of the adaptive threshold, λ≥0 controls the ambiguity-dependent adjustment, and clip(a,l,u)=min(u,max(l,a)). All else being equal, increasing λ does not lower the threshold for the corresponding pixel, while continuous weights allow the attenuation to vary gradually; however, this mechanism does not guarantee the preservation of all weak responses.

For continuous filtering, the retention confidence and adaptive threshold define the weight:

(25) W(i)=σ(α[p(i)−τ(i)]),α>0,

where the positive parameter α controls the transition steepness. Responses well above the threshold receive weights near one, responses well below it receive weights near zero, and boundary responses are attenuated continuously. The confidence-derived ambiguity proxy satisfies $U(i)=1-[2p(i)-1{]}^{2}\in [0,1]$, attaining its maximum at p(i)=0.5. For finite α>0, the continuous weight satisfies 0<W(i)<1, with W(i)=0.5 when p(i)=τ(i). The threshold bounds were $\tau_{min}=0.35$ and $\tau_{max}=0.65$, with an ambiguity-adjustment coefficient of λ=0.10 and a transition steepness of α=20. When C-ATG was disabled, a fixed threshold of τ(i)=0.50 was used.

The suppressed microwave image is:

(26) $$I_{mw}^{sup}=I_{mw}⊙W.$$

The spatial weight map acts on the original microwave-response map; the two-channel tensor X is used only inside the segmentation network.

A binary hard-gating special case is:

(27) $$M_{ret}\left(i\right)=I\left[p\left(i\right)\geq \tau \left(i\right)\right],{I_{mw}}^{sup}=I_{mw}⊙M_{ret}.$$

**Figure 8:**
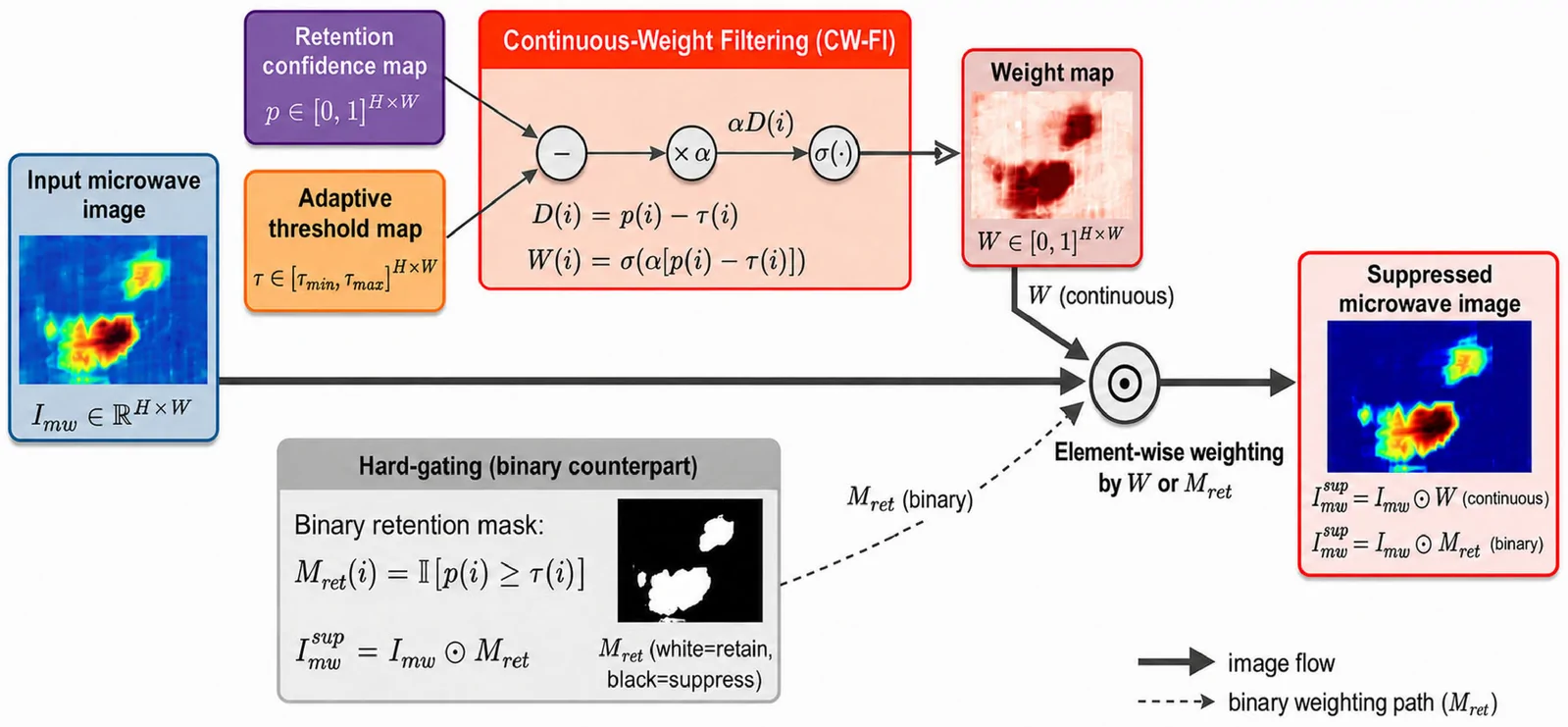
Continuous and binary retention weighting.

The framework therefore defines continuous weighting and binary hard gating within a common notation, as illustrated in Figure 8. 

### 3.5. Training Objective

Let $y_{c}(i)$ denote the one-hot pixel label, and let $m_{i}=y_{1}(i)$ denote the ground-truth retention label. The positive constant $\varepsilon_{log}$ stabilizes the logarithms. The segmentation branch is optimized using the two-class pixel-wise cross-entropy:

(28) $$L_{seg}=-\frac{1}{\left|\Omega \right|}\sum_{i\in \Omega }\sum_{c=0}^{1}y_{c}(i)log(P_{c}(i)+\varepsilon_{log}).$$

To provide a direct gradient to the adaptive-threshold branch through the continuous weight W(i), the per-pixel gate loss is defined as:

(29) $$l_{gate}\left(i\right)=-m_{i}log\left(W\left(i\right)+\varepsilon_{log}\right)-\left(1-m_{i}\right)log\left(1-W\left(i\right)+\varepsilon_{log}\right).$$

The gate loss is averaged over all pixels and combined with the segmentation loss using equal weighting:

(30) $$L_{gate}=\frac{1}{\left|\Omega \right|}\sum_{i\in \Omega }l_{gate}(i),L=L_{seg}+L_{gate}.$$

The differentiable continuous gate is used as the training surrogate whenever the adaptive-threshold branch is learned, so that the gate loss supplies a gradient to that branch. During evaluation and detector-input generation, configurations containing CW-FI retain the continuous output of Equation (26), whereas configurations without CW-FI replace it with the binary mask defined by Equation (27).

## 4. Experiments

The evaluation examines optimizer and learning-rate selection, qualitative suppression behavior, controlled class-wise detection changes, and component ablation using the dataset, acquisition configuration, and detector setup described below.

### 4.1. Experimental Setup and Comparison Scope

Both the front-end segmentation network and the downstream detector were implemented in PyTorch 2.0 and evaluated on an AutoDL platform equipped with a 40-core Intel Xeon Platinum 8458P CPU, 200 GB of RAM, and two NVIDIA H800 GPUs. YOLOv4-tiny with an input resolution of 416 × 416 was used as the downstream detector to evaluate the effect of front-end suppression on damage detection. The detector was trained using the Adam optimizer [28] for 200 epochs with a batch size of 32. The initial learning rates for the two training stages were 0.001 and 0.0001, and the NMS IoU threshold was 0.4. Within each comparison pair, separate detector instances were trained using raw and suppressed inputs, with identical initialization settings, network architecture, training-stage definitions, learning-rate scheduling, and post-processing settings.

The composite backbone in Pair B comprised a deepened CSPDarknet53-tiny main network and a VGG16 auxiliary network. At each spatial resolution of 52 × 52, 26 × 26, and 13 × 13, corresponding feature maps from the two networks were concatenated along the channel dimension and fused using a 1 × 1 convolution. Mish activation was used in the convolutional and residual modules of the main network. For transfer learning, the detection network was initialized with compatible parameters from a model pretrained on PASCAL VOC 2007 and was subsequently trained on the training subset of this study. Anchor-box clustering assigned annotated bounding boxes to clusters based on the maximum IoU and updated each cluster center using the mean width and height of the boxes assigned to that cluster. Label smoothing provided regularization by softening the target class labels. The learning-rate schedule combined cosine decay with warm restarts. Detector-side data augmentation included random scaling, rotation, flipping, and four-image Mosaic, without random photometric perturbations. Geometric transformations were applied consistently to each microwave image and its annotations.

mIoU quantifies mean pixel-level agreement across the clutter and retained-response classes; downstream detector utility is evaluated separately using class-wise AP@0.50.

### 4.2. Optimizer and Learning-Rate Selection

For the front-end segmentation network, four optimizers—stochastic gradient descent (SGD), RMSprop, SGD with momentum, and Adam—were evaluated at the listed initial learning rates. Table 1 reports validation-set mIoU used to select the optimizer and initial learning rate.

Across the validation sweep, mIoU increased as the learning rate decreased from 1 × 10^−3^ to 1 × 10^−4^ and declined at smaller values for each optimizer. These values are used only for within-validation hyperparameter selection and do not establish convergence stability or statistical superiority.

The highest validation mIoU reported in Table 1 is used solely for configuration selection within the current candidate set; there is no guarantee that this configuration remains optimal on unseen data. Repeatedly selecting configurations based on the same validation set may lead to overfitting to that set; therefore, test performance is presented separately in Section 4.5.

### 4.3. Qualitative Analysis of Suppressed Responses

**Figure 9:**
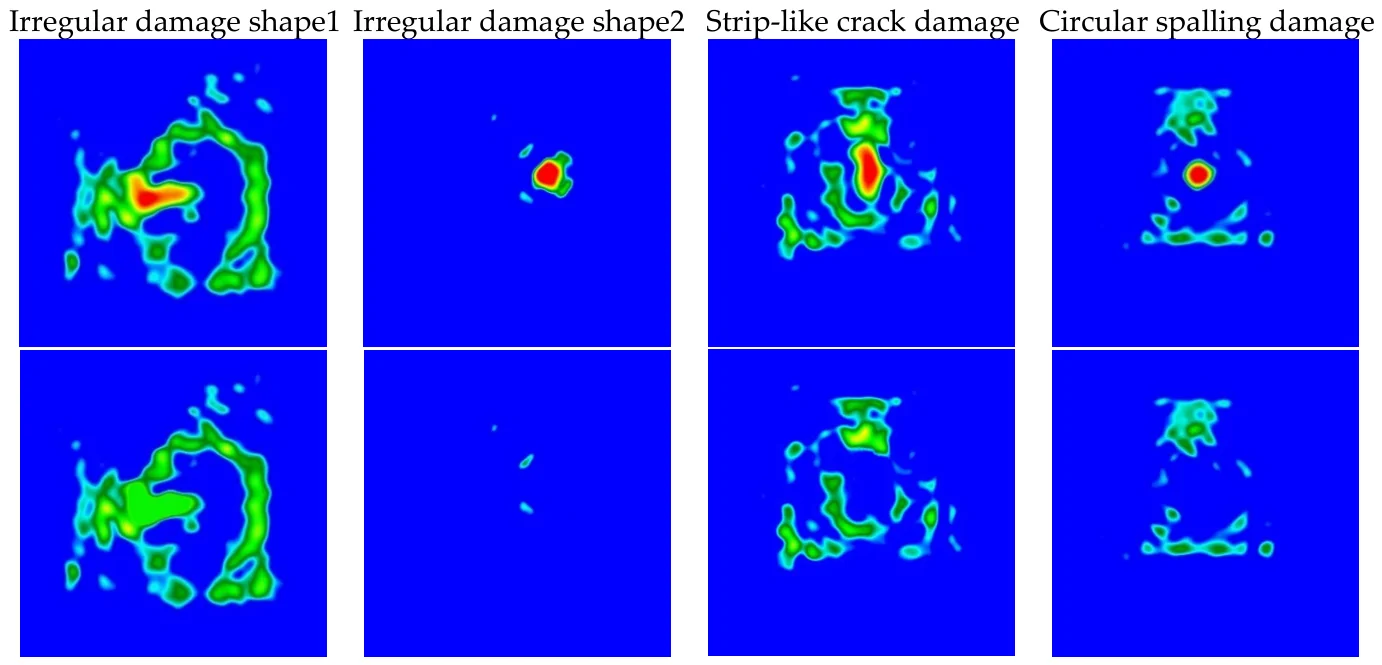
Qualitative suppression results for representative samples. The top row shows the raw microwave-response maps, and the bottom row shows the continuously weighted outputs. The terms ring-like, patch-like, point-like, and band-like describe response morphology rather than establish physical origin. Clutter is interpreted according to the annotation-based definition in Section 2.2.

Figure 9 compares representative raw microwave-response maps in the top row with their continuously weighted outputs in the bottom row. The examples show spatially selective attenuation around ring-like, patch-like, point-like, and band-like response structures rather than uniform amplitude scaling; quantitative segmentation and detection effects are reported in Table 2 and Table 3.

Figure 9 illustrates the selective attenuation of different spatial responses. This phenomenon is explained qualitatively, and the benefits of damage detection are evaluated through the matched comparisons presented in Table 2.

### 4.4. Detection Change Under Controlled Front-End Suppression

Table 2 presents two matched comparisons on the held-out test subset. Pair A uses the baseline YOLOv4-tiny configuration, whereas Pair B uses an enhanced configuration incorporating CB, Mish, KM, TL, LS, CA, and DA. Details of initialization, learning-rate scheduling, and the implementation of these components are provided in Section 4.1. Within each pair, the raw microwave input is compared with the continuously weighted output defined by Equation (26), and separate detector instances are trained under otherwise identical detector-side settings, as described in Section 4.1.

For Pair A, AP@0.50 increased by 4.18 percentage points for strip-like cracks (83.82% to 88.00%) and by 11.52 percentage points for circular spalling (71.97% to 83.49%). Both sets of matching configurations showed positive changes in category-wise AP, though the magnitude of growth varied depending on the damage category and detector configuration. AP@0.50 provides a composite measure reflecting confidence ranking as well as the impact of false positives, false negatives, and localization matching; while the results support an overall improvement in detection, they do not isolate the dominant factor driving changes in specific error types.

For Pair B, AP@0.50 increased by 6.21 percentage points for strip-like cracks (86.10% to 92.31%) and by 1.90 percentage points for circular spalling (88.01% to 89.91%). The different class-wise gain pattern indicates that the observed benefit depends on both damage category and detector-side configuration.

AP@0.50 improved for both damage categories in both controlled comparison pairs. These results reflect the detection gains observed under the configurations evaluated in this study. Because no repeated-run analysis was performed, they do not establish statistical stability across random runs.

The dataset comprises a total of 6237 annotated damage instances, averaging approximately 3.95 instances per image pair—with cracks averaging about 2.01 per pair—and includes images containing multiple instances. The front-end performs pixel-wise weighting across the entire image without pre-specifying the number of damage instances. AP metrics evaluate the overall detection performance for the annotated instances but are not used to determine the minimum resolvable spacing between adjacent cracks.

Front-end suppression uses the continuously weighted output defined by Equation (26). CB, Mish, KM, TL, LS, CA, and DA denote the composite backbone network, Mish activation function, K-means anchor-box clustering, transfer learning, label smoothing, cosine annealing learning-rate scheduling, and detector-side data augmentation, respectively. The pretraining source, training parameters, and descriptions of the main Pair B components are provided in Section 4.1. Within each comparison pair, the detector configuration is kept identical, with only the application of front-end suppression to the input being varied.

### 4.5. Component Ablation of the Front-End Module

Table 3 evaluates the front-end components on the held-out test subset using the baseline detector configuration of Pair A. All front-end configurations retain the base segmentation network. Disabling SA-PPM replaces adaptive scale aggregation with standard pyramid pooling; disabling C-ATG uses the fixed threshold τ; and disabling CW-FI uses a binary retention mask. In the CW-FI-only configuration, continuous weights are computed from the base segmentation confidence and τ using Equation (31). A separate downstream detector is trained for each front-end configuration under the same Pair A detector-side settings.

(31) $$W\left(i\right)=\sigma \left\{\alpha \left[p\left(i\right)-\tau \right]\right\}.$$

Relative to no suppression, every reported component configuration increases both class-wise AP values. Among single-component rows, CW-FI gives the larger class-wise AP values, whereas C-ATG gives the higher mIoU. This different ranking shows that binary segmentation agreement and downstream detector utility are related but not interchangeable.

As shown in Table 3, the mIoU for the CW-FI single-component configuration is 84.47%—lower than the 85.30% achieved by the C-ATG single-component configuration—yet the AP@0.50 values for cracks and spalling improved by 0.35 and 4.20 percentage points, respectively. The combination of C-ATG and CW-FI yields an mIoU of 84.68% (a decrease of 0.62 percentage points relative to the C-ATG-only setup), while the AP values for the two categories increased by 0.54 and 4.88 percentage points, respectively. Upon incorporating SA-PPM, the full configuration achieves an mIoU of 87.30%, with category AP values of 88.00% and 83.49%.

These results demonstrate that pixel segmentation consistency is not equivalent to downstream detection utility. While mIoU is calculated based on discrete categories prior to thresholding, continuous weights alter response amplitudes in the detection input, allowing pixels near the threshold to retain intermediate intensity values. Consequently, mIoU alone cannot fully characterize the value of the front-end component for instance detection. Given the same probability map and ground truth labels, simply switching between the final binary output and the continuous output does not alter the mIoU as defined in this paper, thus the mIoU variations observed across different configurations in the table should not be attributed to the final weighting operation itself.

Reference [13] studied PSPNet-based clutter suppression using 1448 image pairs and a 9:1 training-to-test split; its Figure 9 shows an input size of 473 × 473 × 3. The present study uses 1577 image pairs, separate training, validation, and test subsets, and a front-end input size of 416 × 416 × 2. The 93.67% mIoU in Reference [13] was reported in an optimizer and learning-rate comparison, for which the evaluation subset was not explicitly identified; the 87.30% mIoU reported here is evaluated on the held-out test subset. These values were obtained under different protocols and do not establish relative performance under matched conditions. The present evaluation therefore relies on within-study component ablations and matched downstream detection comparisons.

## 5. Discussion

Table 2 shows AP@0.50 gains for both damage categories in both comparison pairs. The larger within-pair gain occurs for circular spalling in Pair A and for strip-like cracks in Pair B. These differences indicate that the observed gains depend on the damage category and detector configuration. Front-end weighting changes response amplitudes and local contrast, providing a possible explanation for this dependence. AP@0.50 alone cannot separate the contributions of false positives, false negatives, and localization changes or attribute the different gains to an individual detector-side enhancement.

The ambiguity proxy U reflects only the extent to which a single prediction approaches the binary decision boundary; this value may remain low even when the model makes a confident error. Threshold intervals and continuous weights control the numerical range but are not equivalent to probability calibration, nor do they encompass all sources of uncertainty.

The ranking of mIoU in Table 3 does not fully align with that of class-wise AP, indicating that pixel-level classification metrics alone are insufficient to represent the detection value at the front end. Continuous weights preserve response magnitude hierarchies, whereas mIoU evaluates discrete category consistency; therefore, this paper presents both types of metrics.

The current results are based on RAMDD data acquired via a 4–18 GHz near-field multi-probe system and two configurations utilizing YOLOv4-tiny; the extent of gain for other single-stage or two-stage detectors remains undetermined. Variations in operating frequency, material composition, and curvature may alter response scales, amplitude-phase distributions, and local incidence conditions, necessitating verification of the applicability of preprocessing methods and model parameters for migrated applications. In terms of computational cost, given the segmentation probability and the base sample threshold, the computational complexity of pixel gating and image-weighting scales linearly with the number of pixels. Generating the final single-channel weighted output from a 416 × 416 input requires 173,056 pointwise multiplications. This count excludes network inference and acquisition reconstruction; therefore, it cannot be used to infer the parameter count, MACs, or real-time performance of the entire pipeline. Consequently, the conclusions of this paper are limited to the relative detection performance associated with front-end suppression under the existing experimental conditions.

Subsequent work will further evaluate transfer performance across different materials and acquisition conditions, damage localization in the vicinity, confidence calibration, and the computational overhead of the entire workflow.

## 6. Conclusions

This paper proposes a front-end method for clutter suppression in microwave images of curved RAM surfaces, integrating dual-channel representation, scale-adaptive aggregation, and confidence-driven continuous weighting. The complete configuration achieved an mIoU of 87.30% on the hold-out test subset and improved AP@0.50 for two defect categories under the matching detection setting, with a maximum gain of 11.52 percentage points. The results demonstrate that input-side weighting enhances defect detection under the evaluated conditions and that pixel classification and instance detection metrics should be used in conjunction.

Introducing continuous retention weights between image reconstruction and detection offers a way to balance interference attenuation with the preservation of defect information. Current validation is limited to the established dataset, visible surface defects, and the specific detector configuration employed; future work will involve validation across different materials and acquisition conditions, analysis of adjacent defect instances, and evaluation of the computational overhead of the entire pipeline to clarify the method’s scope of applicability.

## Figures and Tables

**Table 1:** Validation-set mIoU under different optimizers and initial learning rates (%).

Optimizer	0.001	0.0005	0.0001	0.00005	0.00001
SGD	74.46	79.97	82.30	77.03	75.31
RMSprop	84.31	89.02	90.00	88.33	85.80
SGD with momentum	85.66	89.05	91.20	86.91	84.77
Adam	85.46	90.45	94.91	91.98	87.68

**Table 2:** Class-wise AP@0.50 (%) for raw microwave inputs and continuously weighted outputs under baseline and enhanced YOLOv4-tiny configurations.

Pair	Detector Configuration	Front-End Suppression	AP@0.50, Strip-like Cracks	AP@0.50, Circular Spalling
A0	Baseline detector	No	83.82	71.97
A1	Baseline detector	Yes	88.00	83.49
B0	Enhanced detector (CB + Mish + KM + TL + LS + CA + DA)	No	86.10	88.01
B1	Enhanced detector (CB + Mish + KM + TL + LS + CA + DA)	Yes	92.31	89.91

**Table 3:** Test-set component ablation using mIoU and class-wise AP@0.50 (%).

Method	SA-PPM	C-ATG	CW-FI	mIoU	AP@0.50, Strip-like Cracks	AP@0.50, Circular Spalling
No suppression				N/A	83.82	71.97
SA-PPM only	✓			81.08	86.20	74.10
C-ATG only (hard gating)		✓		85.30	87.10	77.20
CW-FI only (continuous weighting)			✓	84.47	87.45	81.40
C-ATG and CW-FI		✓	✓	84.68	87.64	82.08
SA-PPM, C-ATG, and CW-FI (Full)	✓	✓	✓	87.30	88.00	83.49

Note: mIoU is computed from the pre-gating argmax segmentation labels using Equation (4) and is not applicable to the no-suppression baseline, which has no segmentation output. Configurations with CW-FI apply continuous weighting to the raw microwave-response maps; the other front-end configurations apply binary gating. Separate downstream detectors are trained using the matched settings in Section 4.1. Both mIoU and class-wise AP@0.50 are evaluated on the same held-out test subset.

## Data Availability

The raw/processed data required to reproduce these findings cannot be shared at this time as the data also forms part of an ongoing study.
